# Book Review

**Published:** 1929

**Authors:** 


### Reviews of Books

**The Treatment of the Sick Poor of this Country** (Rogers Prize Essay, 1926). By H. J. McCurrich, M.S., M.B., F.R.C.S. Pp. viii., 132. London: Oxford University Press. 1929. Price 5s.—The wide latitude allowed by the judges of the Joseph Rogers Memorial Prize has been taken full advantage of by Dr. McCurrich. An Essay was asked for, and a captious critic might ask what is the limit of size of an essay. The author has composed enough for a book and was awarded the Prize. Most of the material of this book is a history of the medical service to the poor, taken from many sources and as an historical survey is of interest. We do not find that Dr. McCurrich has evolved any original constructive policy for the service, which is admittedly not fully satisfactory. This, we take it, was the intention of the founder of the Prize, and we fear that those who desire the betterment of the Poor Law medical service will not benefit much by the book. Neither are we always in agreement with the views that Dr. McCurrich expresses on the efficiency of the present state of affairs, but these are his opinions and the solution of some of the difficulties is obscure. Two slight errors have been detected. The Society of Merchants of Bristol never, as a corporate body, equipped or sailed ships from that Port. They acted more as a Harbour Trust, to use a modern term, renting the harbour at a small sum in return for the dock dues and an undertaking to build quays. The other is, the name of Dr. Rogers' more distinguished brother was not Theodore as given in the bibliography, but Thorold.

**Tweedy's Practical Obstetrics**. Sixth Edition. Edited by B. Solomons, M.D., F.R.C.P.I. Pp. xxii., 759. Illustrated. London: Oxford University Press. 1929. Price 25s.—This maintains the high standard of the previous editions, and embodies all the modern methods of practice and treatment associated with the Rotunda Hospital. Changes of opinion in cause and treatment have been indicated in the text, and this is seen in the treatment of accidental hemorrhage. Ante-natal care is not dealt with at much length, but this is made up for in that pelvimetry is very fully considered and well illustrated. The modern theories of toxæmia are all set out, and the very successful treatment adopted at the Rotunda fully described. The text is very readable and the illustrations good and easily understood. It is an excellent text-book.

**Nephritis.** By T. I. BENNETT, M.D., F.R.C.P. Pp. viii., 94. 2 Plates. London: Oxford University Press. 1929. Price 6s.—This monograph embodies the substance of the Goulstonian lectures delivered by Dr. Izod Bennett. The problems of uræmia, œdema and blood-pressure are discussed in the light of recent research in connection with nephritis. The author concludes with a most valuable and practical chapter on the treatment of nephritis, and diet scales and tables for constructing low protein diets are added. The main purpose of the book is a demonstration of the fact that nephritis is a general dyscrasia, and not simply a disease of the kidney. The importance of the extra-mural factor in Bright's disease is as yet very imperfectly appreciated by the medical profession, and this very seriously confuses the problems of prevention, classification and treatment. By his lucid style and skilful marshalling of facts the author has succeeded in infusing fresh interest and new life and shape into the study of this group of renal diseases.

**"Terminal" Disinfection.** By Professor C. CHAGAS. Pp. 45. Edinburgh: W. Hodge & Co. 1928. Price 2s. 6d.—In this excellent little book the Director-General of Public Health for Brazil sets forth clearly the arguments against the employment of terminal disinfection after acute infectious diseases. It must be pointed out that he does not in any way minimise the value of "current" disinfection, *i.e.* disinfection especially of bedclothes and other fomites carried out while the patient is going through the course of his illness. Dr. Chagas's argument is that the life of most pathogenic organisms outside the human body is comparatively short, and that they are probably dead by the time terminal disinfection is carried out at the end of the illness. Certain large towns in America and England have discarded terminal disinfection many years ago, and are perfectly satisfied with the results. The only criticism we would make upon Dr. Chagas's exposition of this theory is that, like a good lawyer, he stresses his own points to their utmost value and pays but little regard to possible counter-arguments. If the matter was quite so well proven as he would lead us to believe, it would not still be the case that the majority of large cities in the world still carry out terminal disinfection after the more serious infectious diseases, adopting as they do the attitude that while there is any doubt it is better to err on the side of safety. An instance of such doubt may be found in the recent discovery by a number of different workers of hæmolytic streptococci in books which have been used by scarlatina patients. Nevertheless, we believe that the main principles laid down in this book are correct, and will probably ultimately become the generally accepted practice, though possibly in a modified form.

**Parkes' and Kenwood's Hygiene and Public Health.** Edited by H. R. KENWOOD, M.B., D.P.H., and H. KERR, M.D., D.P.H. Eighth Edition. Pp. xii., 823. Illustrated. London: H. K. Lewis & Co., Ltd. 1929. Price 21s.—One does not so much expect " Parkes and Kenwood " to follow a recognized standard as look to it to set one up. But no book, however ably it may be written, which deals with a series of activities so flexible and so progressive as those connected with the public health can long survive simply on its reputation. It must, in successive editions, keep up to date, and that is precisely what the eighth edition has done, and done admirably. The revision, as one would expect, bears the stamp of authentic practical knowledge and experience of present-day developments. Not only students, but those engaged in the routine work of a health department, will find the book full of suggestive and helpful information on almost every aspect of their work. Amongst the newer matters dealt with are Maternity and Child Welfare, Industrial, Marine, Tropical and International Hygiene, and the School Medical Service. Adequate descriptions are given of such comparatively recent measures as the Meat Regulations of 1924, the Milk (Special Designations) Order, the Preservatives in Food Regulations, the Puerperal Fever Regulations, and so forth. Some reference might have been made to the possible cerebral complications of vaccination, and perhaps to the Adoption Act, but any discussion on the effects of the new Local Government Act had to be left to the next revision. In a word, this edition has not only maintained, it has enhanced the reputation of what is universally recognized as a standard text book.

**Manson's Tropical Diseases.** Edited by P. H. Manson-Bahr, M.D., D.T.M. & H., F.R.C.P. Ninth Edition, revised. Pp. xx., 921. 35 plates, 401 figures, 6 maps, 34 charts. London: Cassell & Co., Ltd. 1929. Price 31s. 6d.—Medicine and our knowledge of tropical diseases do not stand still. This classic book has already been through eight editions in less than thirty years, each called for by advances in the knowledge of diseases of warm climates, their etiology, diagnosis, course and treatment. This ninth edition, published four years after its predecessor, has its origin from the same cause. In it are set forth many new facts, also changes in our ideas with regard to some diseases, due to recent work. The most notable example is Yellow Fever. Its presence in West Africa has caused much concern of late years, and it has been under investigation for some time past. Special attention has been given to the subject of treatment, and the reader will find that of the various diseases full, complete, and explicit. Naturally, that of Malaria, the most frequent of tropical diseases, receives special attention. That suitable to each of the various types of the disease is pointed out. Special points for special cases are indicated, and where necessary emphasized. The protozoological and laboratory sections in the appendix has been thoroughly revised and brought up to date and a new section inserted on blood transfusion " suitable for employment under tropical conditions." Although some thirty new text figures with five half-tone colour plates have been added, the total number of pages in the book remains practically the same, and it thus retains one of its original aims, is " of handy size." In our opinion this manual of diseases of warm climates is full of sound, reliable and adequate information, and ought to be in the kit of all those who have to deal with tropical diseases.

